# Analyzing the Contents of a Large, Public Online Peer Support Forum for Obsessive-Compulsive Disorder: Thematic Analysis

**DOI:** 10.2196/60899

**Published:** 2025-05-26

**Authors:** Nora Yanyi Sun, Christopher Pittenger, Terence Ching

**Affiliations:** 1 Harvard University, 58 Plympton St, Cambridge, MA, 02138, United States, 1 904-646-8255; 2 Department of Psychiatry, Yale University School of Medicine, New Haven, CT, United States

**Keywords:** obsessive-compulsive symptoms, reassurance-seeking, peer support group, thematic analysis, Reddit, online peer support forum, online support, obsessive disorder, anxiety disorder, debilitating chronic anxiety disorder, anxiety, stress, depression, website, treatment, online forums, obsession, patients

## Abstract

**Background:**

Obsessive-compulsive disorder (OCD) is a debilitating chronic anxiety disorder with low rates of remission. r/OCD is a peer support forum hosted by the Reddit website with over 180,000 users and 100‐200 new posts daily. While peer support has been associated with improved treatment adherence and outcomes, online forums can also be an outlet for performing and accommodating compulsions (eg, seeking and receiving reassurance), which can ultimately exacerbate OCD.

**Objective:**

This study aims to inductively assess what types of content are posted on r/OCD, an online peer support forum for individuals with OCD, to better understand the prevalent topics, dynamics, and norms of such online communities.

**Methods:**

To describe the explicit content of the forum, 132 new posts with a total of 739 comments added to the subreddit within a 24-hour period from August 4, 2023, to August 5, 2023, were manually downloaded and coded using inductive thematic analysis. Though posts beyond the first 24 hours were downloaded, saturation of ideas was observed after the first 110 posts, and thus, no posts after the first 24 hours were included in the analysis. Through inductive thematic analysis, codes were organized into overarching themes.

**Results:**

Four main themes emerged during analysis: (1) validating OCD symptoms (n=69 total posts and comments), (2) connecting with peers with similar OCD themes (n=512), (3) coping with symptoms (n=280), and (4) sharing treatment experiences and advice (n=118). Many posts involved users describing their symptoms, questioning if a particular symptom was OCD, and asking other users if they had similar experiences. Users frequently categorized their OCD into subtypes using abbreviations (eg, “ROCD” [relationship OCD]) to seek others with similar experiences. A minority of posts solicited and provided advice on therapy and medication. Users were supportive and encouraging of each other’s recovery journeys.

**Conclusions:**

Online peer support forum users demonstrated substantial knowledge of OCD and treatments; these forums enable users to receive social support and exchange helpful information and peer experiences related to seeking medical treatment. However, many users use the platform to connect with others experiencing similar subtypes of OCD or to seek confirmation that their symptoms are OCD, which is promoted by community norms and may suggest they are reassurance-seeking. Close moderation by health care professionals and restricting detailed sharing of OCD symptoms to prevent reassurance-seeking may be important for ensuring a net positive impact of online peer support forums for OCD. In addition, clinicians should consider if their patients may be reassurance-seeking on this and similar OCD forums, given their popularity. Future studies should conduct interviews with users and investigate patterns of user ability to further understand the potential harms and benefits of online peer support forums.

## Introduction

### Background

Obsessive-compulsive disorder (OCD) is characterized by recurrent, unwanted, distressing thoughts, images, or impulses termed obsessions. These obsessions are highly diverse and can often be idiosyncratic [1]. In response to obsessional distress, individuals with OCD typically engage in repetitive or highly ritualized physical or mental behaviors aimed at reducing or preventing the distress, termed compulsions [2]. Though highly heterogeneous, a majority of OCD symptoms can be categorized into four dimensions: (1) obsessions about contamination, accompanied with cleaning rituals; (2) obsessions about causing harm, accompanied with checking or reassurance-seeking rituals; (3) obsessions about incompleteness, accompanied with ordering or rearranging rituals; (4) obsessions about morally unacceptable violent, sexual, or religious behaviors, accompanied with mental rituals intended to negate these thoughts [1,3].

OCD is a debilitating condition resulting in significant impairment of quality of life [4] that affects around 2% of adults in the United States, with women being 1.5 times more likely to experience OCD [5]. Current first-line treatments for OCD include cognitive-behavioral therapy using exposure and response prevention (ERP) and pharmacotherapy with selective serotonin reuptake inhibitors [6,7]. Though these therapies are effective, an estimated 57% of people with OCD do not access treatment due to various barriers, such as financial or geographical constraints, a deficit of available therapists, and mental health stigma [8,9].

Peer support groups may encourage and help people with OCD better understand their condition and motivate them to identify, seek, and continue engaging in treatment [10]. Social media is a common platform for social interaction in modern societies, and its use generates a substantial impact on anxiety disorders [11]. While in-person OCD support groups overseen by health care professionals tend to be beneficial for OCD patients [12], such support groups may be expensive or geographically inaccessible for some individuals. However, OCD peer support groups, particularly on Facebook (Meta), may also have mixed or negative effects on patients [13]; thus, the content of these online communities should be further investigated.

An online forum focused on OCD may provide particular benefits. OCD patients may be reluctant to discuss symptoms with physicians due to their taboo subject matter [6], but may be more willing to do so on an anonymous online forum. Interacting with other individuals with OCD in an online community may encourage individuals to recognize and avoid performing compulsions, as well as learn about and seek medical treatments—for example, users may use the platform to exchange helpful information about treatment experiences and assist other users in enrolling in treatments they were previously unaware of.

However, an online forum with OCD may also be harmful in certain circumstances. Misinformed or underqualified users or “trolls” may perpetuate counterproductive or harmful information about OCD or send rude messages in the community. In addition, many individuals with OCD engage in pathological and compulsive reassurance-seeking, which provides temporary relief but ultimately aggravates their condition [14]: for example, an individual with symptoms in the unacceptable thoughts dimension of OCD may use an online forum to confess their OCD obsessions to allay their fears [15]. Symptom accommodation may occur when other users comment to reassure the original poster that they are not a pedophile, that what they fear they did was a false memory, and that such reactions are a symptom of OCD. Although such reassurance provides temporary relief, it ultimately paradoxically reinforces obsessions and prompts future reassurance-seeking and other compulsions [16].

As no research has been done on public Reddit-based forums specifically for OCD, despite their 100s of 1000s of active users, a robust qualitative understanding of the structures and conventions of these forums would be valuable for clinician awareness and provide important foundations for further investigations on potential harms and risks posed by these forums.

### Objective

The primary purpose of this study is to explore what types of content are posted in r/OCD, a large, public online support forum for OCD hosted on Reddit, to build our understanding of the dynamics and norms of such online support communities.

Reddit is a free online social media platform accessible in most nations in the world that enables registered users (Redditors) to add posts and comments that may include text, images, videos, links, and GIFs in a variety of themed communities known as “subreddits.” Reddit, which enables users to post anonymously and hold discussions in the comments, hosts over 100,000 active subreddits on a wide range of topics [17], including psychopathology, in subreddits such as r/Anxiety, r/Depression, and r/OCD. Reddit is predominantly used by men (78%) between the ages of 18 and 29 (59%) [18].

The r/OCD subreddit is an online forum dedicated to users with a specific interest in OCD, including but not limited to individuals living with, or family, friends, and acquaintances of people living with OCD. Posts in this forum are divided into categories, known as flares, including “questions about OCD,” “crisis,” “articles,” “discussion,” and “sharing a win.” The forum is moderated by 6 users, one of whom is a licensed therapist. It is currently the largest public online forum targeting users with OCD, with 180,000 users and 100‐200 new posts added every day, as of June 2023. The usage of this forum, or any other online forum for OCD, has not been previously studied.

Thematic analysis has previously been used to study posted content in other online forums used by individuals with mental illness, such as eating disorder support forums [19], forums dedicated to other potentially taboo topics, such as men’s sexual health [20], and forums focused on response to treatment and care practices, such as tinnitus treatment [21]. Using inductive thematic analysis, we captured the main types of posts and exchanges taking place in the subreddit to better understand what posters use this platform for.

## Methods

### Data Collection

Reddit has a 1000-item listing limit: users can view the 1000 most recent posts and comments within a subreddit. To capture as much data as possible, on August 8, 2023, the lead author (NYS) manually downloaded every post and comment added to the r/OCD subreddit over a 72-hour period, from August 4, 2023, to August 7, 2023. The coders felt that saturation of ideas (defined in Data Analysis) was reached after coding the posts collected over the first 24-hour period; this comprised a total of 132 new posts with a total of 739 comments that were added to the r/OCD subreddit over this period. Similar Reddit content analyses have examined 100‐200 posts [22,23].

### Data Analysis

To most directly capture the content in the subreddit, we conducted inductive thematic analysis using a postpositivist “scientifically descriptive” small-q approach [24]. Analysis was performed inductively at the explicit level—a method used to describe sets of previously uncharacterized data [25]—as there were no suitable pre-existing frameworks characterizing online peer support groups for people with OCD.

The lead author (NYS), a female student and research assistant who has previously led inductive thematic analyses using data from social media, read all posts in the data set and developed an initial codebook focused on characterizing the categories of content being posted (eg, describing a symptom and asking for advice on a medication) rather than the details of the content (eg, individual worries about being a pedophile or having eaten contaminated food). To enhance dependability, the initial codebook, which contained 58 codes, was independently reviewed by the last author (TC), a male licensed clinical psychologist and instructor who was also familiar with the data and had expertise in OCD research and qualitative analytical methods. The 2 authors discussed and reflected on potential oversights and biases until they reached a consensus on the initial codebook.

The lead author coded the data by thread (ie, a post and its comments) in a spreadsheet, with each post or comment being labeled with at least one code. During the coding process, the lead author iteratively discarded codes that were not used, combined codes that were highly similar, and added codes to describe new patterns observed. The last author regularly reviewed coding and discussed any potential biases or overinterpretation of not explicitly present phenomena with the first author until consensus was reached. Saturation of ideas, which was defined as no new codes or themes emerging in new threads [26], was observed within the first 110 posts coded.

After the coding of the entire data set was completed, and agreement on all codes was reached between the first and last author, the coding sheet contained 78 codes. Due to the higher number of resulting codes, both authors agreed to eliminate codes that did not appear a substantial number of times in the data set. The number of times each code was applied was tabulated, and there appeared a clear divide with a cluster of codes that appeared 2‐3 times versus codes that appeared more than 10 times. The codes that appeared less than 10 times were removed from the data set, leaving 38 codes.

Finally, through discussion and a consensual decision-making process [27] between the first and last author, these 38 codes were grouped into overarching themes. The themes were iteratively refined by discarding ones that were too heterogeneous or without enough support from the data, combining ones that were too similar, and so forth, until the authors arrived at a set of 4 themes to capture the text [24].

### Ethical Considerations

All data collected from the r/OCD subreddit were posted publicly online and did not require any permission to access. All posts and comments analyzed in this study were posted by anonymous Reddit users and are not identifiable. This study (IRB Protocol ID: 2000036062) received a “Not Human Subjects Research Determination” from the Yale Institutional Review Board.

## Results

### Overview

We analyzed 132 new posts with a total of 739 comments added to r/OCD, an online support forum focused on OCD, during a 24-hour period. The user-selected category (ie, flare) breakdown of the posts were as follows: 7 were tagged as “Discussion,” 11 as “I just need to vent,” 69 as “I need support,” 30 posts as “Question about OCD,” 6 as “Crisis,” 4 as “Art Film Media,” and 5 as “Sharing a Win!”

During the coding process, we focused on characterizing the types of content being posted, and 38 codes were developed (refer to Data Analysis subsection). The 38 codes were subsequently and consensually organized into 4 main themes that capture the types of discussions taking place in the r/OCD online support forum: (1) validating OCD symptoms (69 total posts and comments), (2) connecting with peers with similar OCD themes (512 total posts and comments), (3) coping with symptoms (280 total posts and comments), and (4) sharing treatment experiences and advice (118 total posts and comments). Posts and comments could be categorized into multiple themes. Quotes provided below are lightly edited for clarity and grammar.

### Theme 1: Validating OCD Symptoms

Under this first theme, which includes 69 posts and comments, Redditors on r/OCD posted descriptions of their own fears and behaviors, and asked the subreddit community whether their behaviors could be caused by or related to OCD. Some Redditors posting content that fell under this theme appeared to be somewhat unfamiliar with OCD, expressing uncertainty about whether their concerns are descriptive of or related to OCD.

**Post title**: Am i a [expletive] pedo?

**Post text**: I was watching Reddit and some baby reacting to a video. My groinal part had a weird feeling then like it moved or something. I do get that out of no where but I’m scared that what if I’m a pedo? Is ocd the cause of this? Please help.

However, many others posting content that fell under this theme seemed familiar with OCD; some mention already having an OCD diagnosis, while others state that they have family members or close friends with OCD. They appeared to be aware that their symptoms were “probably OCD” and/or used terminology associated with OCD to describe their symptoms, but subsequently seemed to express doubt about whether the label is valid. This matches clinical descriptions of “OCD about OCD,” in which individuals worry obsessively about whether their symptoms are truly OCD. While this behavior was likely reassurance-seeking, and it typically elicited reassuring or clarifying responses from others, it was not directly labeled as such.

**Post title**: Scared of getting cold sores

**Post text**: I have pretty intense contamination OCD. Someone I'm interested in gets cold sores. I've never had them myself but I fear them giving me herpes and having to disclose that for the rest of my life. I can't tell if its just my OCD, or if im thinking rationally and it’s my OCD just dramatizing it? I know that you can pass it without having an outbreak which is what has me spiraling. Should I cut things off? I really don't want to

**Post title**: Is this even an obsession?

**Post text**: I am obsessed over thinking about whether people hate me and want the worst for me and then I try and go ask for reassurance. And then I do it all over again.

In response to these posts, other Reddit users commented on whether they believed the OP has OCD. In most posts, users affirmed that the individual has OCD. These responses often overlapped with theme 2 (connecting with peers with similar OCD themes); after agreeing that the OP’s experience was a symptom of OCD, they would detail their own experience with a similar symptom. In very few cases, Reddit users suggested that the symptom was normal or attributed it to another diagnosis.

**Comment**: Yes [this is a symptom of OCD]!! It’s called magical thinking in OCD land

### Theme 2: Connecting With Peers With Similar OCD Themes

Connecting over shared experiences related to OCD was by far the most common type of post or comment that appeared in r/OCD, with a total of 512 posts and comments. Shared experiences could include obsessions, compulsions, and triggers, as well as frustrations relating to OCD. Causal attributions for OCD were also common, concerning events or experiences in users’ youth. Most users who posted content falling under this theme appeared somewhat familiar with OCD. In almost all cases, individuals appeared to understand what their obsessions and compulsions were; they correctly used medical terms, such as “groinal responses,” to describe their experiences.

Furthermore, there are common abbreviations on the forum that these users consistently use, such as “ROCD” (relationship OCD, or obsessive-compulsive concerns related to one’s personal relationships), “POCD” (pedophilia-themed OCD, or obsessive doubt about whether one is a pedophile). Interestingly, the term “Pure O” (compulsions that are primarily or exclusively mental, which are often mistaken as obsessions) was also used frequently and erroneously [28]. This and the other terms do not always perfectly correspond to the scientific literature’s understanding of OCD symptom dimensions; rather, they seem to be popularized by the r/OCD community, within the community itself.

Sometimes, the user used strongly emotional language, venting about symptoms, and detailing how OCD was “ruining [their] life,” but more often, the user appeared to be succinctly and calmly recalling their experiences.

Finally, this type of content also often ended with the Reddit user asking if anyone else had a similar experience to them, as an attempt at seeking validation from other users. While this behavior was likely reassurance-seeking, it was not directly coded as such, as in some cases, individuals appeared to be seeking individuals who related to their symptoms to vent to, and it is impossible to distinguish from a single post whether individuals were seeking reassurance, searching for peer support, or both.

**Post title**: Fear of being delusional

**Post text**: Hey guys! I have pure ocd for 5 years and mostly my main theme is guess what becoming a schizophrenic. Couple years ago i read here about fear of being delusional. And now i also got this i have weird intrusive thoughts-what if my parents dont love or what if the food is poisoned. I dont believe in this thoughts but i am afraid of them they make my a little of anxious. I am afraid what if i will one day become delusional. I must say that i havent got this fear since 2020. I mostly have other type of schiz theme. Yesterday i was bored and now because my brain cant be calm it suddenly repeat this fear from 2020. Any similar experiences? Thanks :))

Oftentimes, other users replied to their post or comment, relating to their symptoms, triggers, and frustrations.

**Comment**: I can totally relate. I have an extreme phobia about sending horrible messages to everyone in my contacts or downloading very illegal content on my devices when I am not conscious. I also had a long-time suspicion that devices were recording me so I would need to constantly make sure they were totally turned off, closed, or placed face down when not using them. This really got on my nerves. I honestly don't know the cause of this either, but I feel like false memory OCD attacks our values.

This type of content was not limited to OCD-specific symptoms, but also involved expressing frustrations regarding public perception and misconceptions of OCD. Among the most popular threads in the forum were posts that discussed misconceptions related to OCD, bringing many users into the comment section to vent.

**Comment**: I saw a post on Instagram (Meta) recently that was a response to a person who posted a video that was saying intrusive thoughts are your intuition.

**Reply**: well guess I'm a future serial killer because I saw a dead possum. And I'm an irredeemable monster worse than hilter because my room was messy. Man, Ugh, the idea of intrusive thoughts being intuition and being you is horrible.

Reddit users also shared their OCD experiences through referencing art and media, such as songs they found expressed the anxiety they experienced due to their OCD.

**Post title**: Has anybody watched monk

**Post text**: I've been watching it on peacock and I absolutely love it. Idk [if] that’s a[n] accurate representation of some people with ocd either way I relate to him so much when it comes to my own problems.

Some Reddit users created art of their own to share with the community, such as writing out abstract metaphors that convey their experience of OCD.

**Comment**: OCD being egodystonic is why it’s such a torture. It’s like, I'm battling an alternate universe version of myself inside my mind.

### Theme 3: Sharing Adaptive and Maladaptive Coping Mechanisms

Under the third theme, which includes 280 posts and comments, Reddit users described and recommended using a variety of both adaptive and maladaptive coping mechanisms to deal with OCD symptoms on their own. Much of this content took the form of a user describing their obsessions and compulsions (overlapping with theme 2) and asking for advice on how to alleviate their symptoms.

**Post title**: How do you just not let your intrusive thoughts bother you?

**Post text**: I'm really sick of my thoughts and it doesn't help that I suffer from POCD and real event OCD. Sometimes it bothers me but I just ignore it and sometimes it bothers me too much to ignore it. I definitely think I should get therapy but I'm scared to tell my parents that I need therapy. So in the meantime, what are some tips and tricks to not let the thoughts bother you?

Other users then suggested coping mechanisms, sometimes describing their own experiences applying them.

**Comment**: I just started doing this recently after another bad night of intrusive thoughts. I was getting extremely frustrated because I couldn't sleep, so I came up with this "game" to do in my head. I don't really have a name for it. What I do is choose a topic. Literally any topic, like fruits & vegetables, animals, rabbit breeds, TV shows, anything that I can make a list of. Then, I try to name something for every letter of the alphabet. So if I'm doing fruits and vegetables, I go: apple, broccoli, cucumber, date, eggplant... And if I get stuck on a letter, I try to think about it as much as possible. If the intrusive thoughts try to come in, I think to myself, "Not now, I'm trying to think of something that starts with this letter. " If I finally come up with something, I restart the alphabet and work my way up to Z. If I can't think of anything for that letter, I just go to the next letter and continue on. Once I finish that topic, I start a new one. I finished the food one, so now I'm going to make a list of animals. Then I start: alpaca, bunny, capybara, deer, elephant... I keep doing this game either until my head stops buzzing or I pass the hell out in the middle of it. It has helped me so much, and I am a lot calmer now because of it. Apologies if this isn't helpful. It really helps me out so I figured I would share:)

Some of these coping mechanisms are in line with clinical practice and appear to be suggested by individuals who are well-informed about OCD and the nature of obsessions and compulsions. Users commonly suggested recognizing thoughts as being a symptom of OCD, avoiding performing compulsions, and exposing oneself to their fears. Users also often linked resources, such as blogs or YouTube (Google LLC) channels, that offer coaching for individuals with OCD.

**Comment**: I found this article by Anxiety Canada [link] helpful. Scroll down past the diagram – there are a few things you could start trying.

Other coping mechanisms, however, may have inadvertently reinforced the original poster’s OCD symptoms. Users frequently and perhaps unknowingly offered reassurance in their comments, stating that the original poster’s fears will not come true. In some cases, this was done by users who appeared well-informed about OCD and had linked to high-quality clinical resources as well, making it appear that users may not be aware that they are offering reassurance.

The forum is partially moderated by a licensed therapist, who occasionally alerts a user that they are giving harmful reassurance and removes that user’s comment, but due to the high volume of traffic received by this forum, most reassurance-giving comments are not removed. Users also sometimes suggested avoidance techniques, such as avoiding a dirty location if an individual has OCD obsessions relating to contamination, which is a maladaptive response that serves to reinforce the individual’s obsessive fears [29].

**Comment**: I think you should go to the doctor just in case given how you've described the bite. Ik it’s reinforcing OCD but in this one particular situation I think it’s important to make sure [that you don’t have rabies, which is the original poster’s OCD fear].

In addition to sharing coping mechanisms, many users also expressed sympathy and encouragement for other users, providing social support to each other. In return, the users expressed gratitude for the resources and advice offered to them to cope with their symptoms. A similar supportive social culture has been observed on other subreddit communities for individuals battling addiction [30].

**Comment**: Are you talking about Acceptance and Commitment therapy? I am reading about self compassion in relation to therapy and I love the concepts. Thank you for your comment!

### Theme 4: Sharing Treatment Experiences and Advice

Under the fourth theme, which includes 118 posts and comments, Reddit users shared their experiences in and advice regarding a variety of professional treatments, including ERP therapies and cognitive-behavioral therapies, and taking various prescribed medications. Some users did so unprompted, while others did so in response to users asking about others’ experiences with certain treatments that they were considering for themselves.

**Post title**: How do I get over my OCD regarding public toilet fumes?

**Post text:** All this started over quarantine; I never used to get overly OCD until I started watching those videos about germs. It hasn't gone away since then, and it’s gotten to the point where I can barely use toilets in my own home. It seems to be naturally going away a bit but I still get extreme anxiety when it comes to using public restrooms because of the toilet flushing fumes, and the fact that there’s no toilet cover. And help?

Some users used the platform to express criticism of their psychiatrist or therapist, and asked others for advice on whether to stop or change their treatment routine. The most common criticism involved their therapist not seeming to understand how to treat OCD, or concerns about side effects of medication.

**Post title**: Clomipramine and weight gain

**Post text**: Hello everyone I hope you all are doing okay, I just got prescribed anafranil (clomipramine) for ocd I had ocd since I was a kid I just didn’t know that it was a disorder but it got really bad as I grew up, I’ve been on sertraline for three months now and I just got prescribed anafranil and I want to know how does it affect weight gain is it just by increasing your appetite or does it do something to your body that make you produce more fat?

Other users used the platform to describe improvements in their OCD following treatment and offer encouragement to others that it is possible to get better. This content was rare but typically well-met, with many users congratulating the original poster on their progress.

**Post title**: Ate food that made me anxious. I feel sick to my stomach, but also accomplished.

**Post text**: A few days ago, my neighborhood had an unexpected power outage (no storm or anything to indicate it was going to happen, there was just a bang and everything was off) for a little over four hours. I have major anxiety about food going bad – when I am at the grocery, I put refrigerated food into an insulated bag and keep it in the bag until I am at home putting the groceries away and I still feel like the food has gone bad. So, the power going out and cutting power to the freezers and fridge is like my worst nightmare. It’s caused a lot of stress to both myself and my family the past few days because they don't think it’s any problem and think I'm worried about nothing. Now, I know that logically, the stuff in the fridge could maybe have gone bad, but the stuff in the freezer should be totally fine. Still, I have been so stressed about eating it. Just now I warmed up something that was in the chest freezer and ate it. I honestly feel like I'm going to vomit, but I did it and I know that everything is going to be okay.

## Discussion

### Principal Findings and Comparison With Previous Work

To our knowledge, this study is the first study specific to a Reddit forum dedicated to supporting individuals with OCD. OCD is a debilitating disorder, and less than half of people with OCD are able to access medical treatments, such as ERP [8,9]. Online support communities may provide a free and more accessible pathway for receiving social support and valuable information on OCD treatments; however, they may also provide an outlet for individuals with OCD to receive misinformation and seek reassurance anonymously online from other users, aggravating their condition.

We examined the content of the subreddit r/OCD, the largest online support community for individuals with OCD, using thematic analysis. This research extends previous studies that have examined in-person support groups for individuals with OCD [13,31] by exploring a potential internet-based, highly accessible support group. This study draws upon the popular and highly active pre-existing r/OCD subreddit with the goal of providing generalizable observations on the dynamics and norms of online support forums for OCD.

In the most common type of post on this subreddit, users described OCD symptoms, and either inquired whether their symptoms could be classified as OCD and asked if anyone else had had similar experiences and asked other users to suggest coping mechanisms. Then, other users indirectly or directly affirmed that this was a symptom of OCD and shared their own experiences with similar symptoms and/or a combination of beneficial and potentially harmful coping mechanisms for these symptoms.

Subreddit users frequently titled their posts with a subtype of OCD (eg, pedophilia, relationship, sexuality, and cleanliness) based on the theme of the obsessions, which enabled users to rapidly connect with other individuals suffering from the same theme. Notably, “Pure O” was commonly perpetuated by users as a subtype of OCD “without compulsions”—in reality, the compulsions are simply purely mental—suggesting that the subreddit is vulnerable to the spread of misinformation.

While these linguistic features suggested that the user base is overall familiar with OCD, users often expressed lingering doubt about whether their symptoms were due to OCD. Many users made posts asking other users if their symptoms are “probably due to OCD, right?” Previous studies have found that a major motivation that drives participants to join health-related peer support groups is to seek more information on their condition. Users asking these questions may become dissatisfied by information they judge to be of poor quality, which can lead to extensive mental review of their symptoms and the information provided, and ultimately generate greater worry and obsessions about their disorder [13]. Indeed, a previous study by Tan et al [13] examined 10 Facebook groups and 3 Reddit forums and found that internal preoccupation with symptoms was a significant predictor of negative experiences in these groups.

Users’ desire to frequently question their symptoms online is described by the emerging concept of cyberchondria: compulsive internet-checking behaviors of individuals with health-related obsessions due to intolerance of uncertainty, a core trait associated with OCD [13,32].

Though the r/OCD user base demonstrated substantial knowledge about common OCD therapies, such as ERP, users troublingly failed to refrain from or recognize and call out potentially reassuring messages to other users. This resembles the phenomenon of symptom accommodation, in which individuals, especially family members, provide reassurance or otherwise assist an individual with OCD in performing their compulsions with the goal of offering temporary relief, oftentimes despite being aware that this behavior is deleterious [33]. Symptom accommodation has been shown to delay and decrease treatment response [34]; thus, interactions on this widely and frequently visited forum may present a previously undocumented cause for poorer treatment outcomes among individuals with OCD. This counterproductive behavior is unfortunately condoned by the forum moderators: though the subreddit guidelines explain that reassurance-seeking is counterproductive, this guideline begins by stating that “some reassurance is allowed” (as of December 2024).

The forum norm of using subtypes of OCD to connect with other users with very similar experiences may make it especially difficult for users to refrain from accommodating reassurance-seeking, as the community is structured for users to easily find other users whose experiences they may most easily empathize with. In a study of patients’ caregivers, empathy with the patient with OCD was suggested as a driving factor for the caregivers (often family members) who understood that reassurance-seeking was unhelpful in the context of OCD, to provide reassurance to the patient anyway [35].

A smaller number of posts shared therapy, medication, and recovery experiences. Users successfully established camaraderie with other users, shared useful resources on treatment options, and bonded over shared symptom experiences, provided sympathy and encouragement for each other’s recovery, and expressed gratitude. This positive atmosphere was ubiquitous, no matter the type of post or dimension of symptoms being discussed. Past studies have found that individuals with OCD have lower perceived social support [36], but that social support is positively associated with symptom improvement [37] and negatively associated with symptom severity [38]. Platforms such as this one may be an ideal way for isolated individuals affected with OCD to obtain peer support.

Observations of the community’s supportive culture is in agreement with a previous study on a subreddit for opioid addiction recovery, which also found many instances of social support and mutual aid [30], as well as a study of in-person group therapy for individuals with OCD that found that the participants naturally formed their own support group to support each other and exchange recovery strategies [31]. However, our findings contradict a previous study of online peer support groups for individuals with obsessive-compulsive and related disorders, which focused primarily on online trichotillomania peer support groups on Facebook; this work found that participants had a variety of negative social experiences, including cyberbullying and being blocked by the group. This difference may be due to the fact that Facebook groups are smaller and more personal, whereas Reddit is anonymous, and this OCD forum consists of over 180,000 users who seem to visit the forum as needed, making cyberbullying less feasible.

Overall, community norms appear to promote seeking out other users with similar OCD subtypes and experiences and asking for confirmation that certain symptoms are considered OCD. The community culture of reassurance-seeking emphasizes the importance of having knowledgeable moderators on online support forums. While a few reassurance-providing comments were removed by the licensed therapist moderator with an explanation of why offering reassurance is harmful, the volume of posts and comments was too large for a single moderator to parse. However, though a minority of posts discussed treatment-seeking, these posts appeared highly productive for users, who received helpful information and encouragement on their recovery journeys.

A potential improvement to the forum may be restricting the detailed sharing and discussion of specific themes and symptoms or limiting the discussion of specific OCD subtypes, so reassurance cannot be offered. Along this line, the forum can be restructured to promote the exchanging of treatment information, as those posts appeared to be the most beneficial for users, but only represented a minority of content posted. Currently, none of the flairs are dedicated to seeking and receiving advice about medical treatment—creating flairs, such as “Treatment–Medication” and “Treatment–Therapy,” may encourage users to share such posts.

### Strengths and Limitations

This study has several strengths—it examines a large volume of qualitative data from a very well-visited subreddit with over 180,000 users and 100‐200 new posts daily. Individuals from any demographic background may contribute anonymously, and thus the data is less vulnerable to some forms of stigma or underrepresentation compared to other studies that do not use naturally occurring data. Overall, this study provides an important basic understanding of the content posted on this community, which future studies may build off of. As no prior studies have focused on such an online forum despite its large user base, an understanding of the content of such forums is valuable to clinicians, whose patients may be visitors or participants in the forum.

However, study results must be considered in the context of their limitations. First, the goal of this study is to understand the forum’s community rather than individual users; thus, it was beyond the scope of this preliminary study to determine through interviews and user analytics whether most users are first-time visitors or recurring, if users used the platform to seek reassurance, if users truly benefited from exchanging treatment advice, etc. Second, thematic analysis cannot quantify the extent of benefit or harm provided in Reddit forums, which should be further investigated through quantitative analyses that were beyond the scope of this study. Third, while anonymous platforms, such as Reddit, enable users to discuss potentially taboo OCD symptoms with decreased fear of repercussions, they also prevent the collection of user demographics. As noted as a limitation in similar studies, Reddit users are primarily male young adults and may not be representative of all patients with OCD [39]. Fourth, only English posts were included in this study. Thus, the themes identified may not reflect the experiences of users in OCD online support forums hosted on other platforms or in other languages.

### Future Directions

To build upon the results of this study and address the aforementioned limitations, future studies may analyze the usage patterns of frequent visitors to the subreddit (ie, characterizing what type of posts and comments individuals who may be compulsively returning to this subreddit are making) to provide clinicians with better tools to determine the impact OCD patients’ subreddit use may have on their treatment course–whether online forums are helping or delaying it. Future studies may also interview and quantitatively survey subreddit visitors, especially those who make posts that appear to be reassurance-seeking, to assess at the user-level the benefits and harms of visiting the subreddit.

### Conclusions

In conclusion, we observed that most posts on r/OCD involve users sharing their OCD symptoms, finding others with similar symptoms, and potentially seeking reassurance that their symptoms are OCD; forum norms promote using OCD subtypes to seek users with similar experiences. Secondarily, forums are used to exchange information about treatment and provide emotional and social support on each other’s recovery journeys. Overall, our study suggests that individuals with OCD may benefit from participating in online support forums to receive valuable information and social support, but that such forums benefit from moderators with health care backgrounds to prevent establishing a norm of symptom accommodation. Given the over 180,000 users and hundreds of daily new posts every day, we recommend that clinicians should cautiously evaluate their patients’ use of this subreddit or similar internet-based OCD forums to determine if patients may be displaying this pattern of reassurance-seeking.

## Abbreviations

OCD: obsessive-compulsive disorder
ERP: exposure and response prevention
